# Identification of a novel thrombospondin-related anonymous protein (BoTRAP2) from *Babesia orientalis*

**DOI:** 10.1186/s13071-019-3457-0

**Published:** 2019-05-03

**Authors:** Xueyan Zhan, Junwei He, Long Yu, Qin Liu, Yali Sun, Zheng Nie, Jiaying Guo, Yangnan Zhao, Muxiao Li, Xiaoying Luo, Lan He, Junlong Zhao

**Affiliations:** 1 State Key Laboratory of Agricultural Microbiology, College of Veterinary Medicine, Huazhong Agricultural University, Wuhan, 430070 Hubei China; 2 Key Laboratory of Preventive Veterinary Medicine in Hubei Province, Wuhan, 430070 Hubei China; 3 Key Laboratory of Animal Epidemical Disease and Infectious Zoonoses, Ministry of Agriculture, Huazhong Agricultural University, Wuhan, 430070 Hubei China

**Keywords:** *Babesia orientalis*, Thrombospondin-related anonymous protein 2, Microneme protein, Babesiosis

## Abstract

**Background:**

The thrombospondin-related anonymous protein (TRAP) was first discovered in the sporozoite of *Plasmodium falciparum* and TRAP family proteins are secreted by micronemes and transported to the parasite surface to participate in the invasion process. Various TRAP proteins have been identified in apicomplexan protozoans, but there have been few reports about TRAP proteins in *Babesia orientalis*.

**Methods:**

The functional domain of TRAP2 in *B. orientalis* was cloned, sequenced, characterized and compared to the TRAP sequences of related apicomplexan parasites. The functional domain of BoTRAP2 was truncated, named BoTRAP2-1, and then cloned into the pET-28a expression vector. Rabbit anti-rBoTRAP2-1 polyclonal antibody was produced by immunizing three rabbits. Western blot analysis was used to identify the native form and immunogenicity of BoTRAP2. The localization of BoTRAP2 was identified by indirect fluorescence assay (IFA).

**Results:**

The amplified genes of BoTRAP2 are 2817 bp in length, encoding a functional domain of about 938 aa with two vWFA domains, one TSP domain and one transmembrane domain. The amino acid sequence of BoTRAP2 has a high similarity with that of *B. bovis* and *B. gibsoni*. The predicted tertiary structure of truncated BoTRAP2-1 confirmed that BoTRAP2 contains two vWFA domains and a TSP domain, the main functional areas of the protein. The native BoTRAP2 was identified from *B. orientalis* lysate by using rabbit polyclonal anti-rBoTRAP2-1. A band corresponding to rBoTRAP2-1 was detected by reaction with serum from a *B. orientalis*-infected water buffalo, indicating that the protein has a high immunogenicity. IFA showed that BoTRAP2 is mainly localized on the apical end of parasites by rabbit anti-rBoTRAP2-1 polyclonal serum.

**Conclusions:**

The rBoTRAP2 could differentiate serum from *B. orientalis*-infected water buffalo and normal water buffalo, implicating that BoTRAP2 has high immunogenicity and could serve as a candidate antigen for diagnosis of *B. orientalis* infection in buffalo.

**Electronic supplementary material:**

The online version of this article (10.1186/s13071-019-3457-0) contains supplementary material, which is available to authorized users.

## Background

Apicomplexa is a phylum of unicellular eukaryotes with specialized intracellular parasites, including more than 5000 species, some of which are clinically important pathogens such as *Plasmodium*, *Toxoplasma gondii*, *Cryptosporidium*, *Eimeria*, *Coccidia*, *Neospora caninum*, *Babesia* and others, which pose great harm to animal husbandry and public health. *Babesia* is a serious pathogen that infects erythrocytes of vertebrates, causing babesiosis, transmitted by ticks. The main pathogenic mechanisms are hemolysis anemia and organ failure caused by the lysis of infected red blood cells (RBCs), which can lead to organ failure or even death in severe cases. In an *in vitro* culture experiment, a new species termed as *B. orientalis* was found to be very different from other *Babesia* spp. in its morphology, transmission, molecular taxonomy and pathogenicity [1, 2]. Specifically, *B. orientalis* is a hemoprotozoan transmitted by *Rhipicephalus haemaphysaloides*, causing babesiosis in water buffalo mainly in the central and southern parts of China. It can change the structure and function of erythrocytes in host animals, giving rise to the clinical symptoms such as fever, anemia, jaundice, hemoglobinuria and death. Furthermore, there is no vaccine or drug to prevent this disease [3, 4].

Erythrocyte invasion is a prerequisite for the growth and propagation of *Babesia*, and the invasion mechanism by *Babesia* is considered to be similar to that of other apicomplexans, featuring an active and continuous invasion process. For example, when *B. bovis* invades the host erythrocyte, merozoites first adhere to the RBCs, followed by specific site recognition, invasion, and then asexual multiplication in the invaded RBCs [5]. All apicomplexan protozoan invasions of host cells are accomplished through matrix-dependent movement, with the force of this movement consisting of actin-myosin complexes located between the parasite’s plasma membrane and endometrium [6]. The movement complex is formed when a transmembrane protein is attached to a receptor located in the surface of the host cell. The thrombospondin-related anonymous protein (TRAP) family secreted by micronemes are important transmembrane proteins [7, 8], which include one or more thrombospondin type-I repeat domain (TSR), von Willebrand factor A-like domain (vWFA), transmembrane domain (TMD) and cytoplasmic tail domain (CTD) [9]. The location of the TRAP family is essential for invasion of the parasite [10]. The contact between parasite and host cell triggers the release of TRAP from the micronemes to the surface of sporozoite and accumulates at the front to mediate the invasion in combination with the receptor of the host cell. Most TRAP family proteins have an integrin-like vWFA domain and a TSR domain which a parasite uses to bind the erythrocyte surface receptors, polysaccharides and phospholipid-like molecules [11, 12].

In view of the localization and structural characteristics of these proteins, the TRAP family has become a key protein for the invasion of the apicomplexan protozoa. In recent years, it has become a popular molecule for investigating the infection and invasion between apicomplexan protozoa and their hosts. The TRAP proteins that have been successfully identified include *Pf*TRAP protein, TgMIC2, EtMIC1, EmTFP250, BbTRAP2, Bgp18, NcMIC2 and NcMIC2-like 1 [13–19]. In *B. bovis*, there are four TRAP genes named BbTRAP1-4 (XM_001609738, XM_001609762, XM_001609736 and XM_001609760) [20]. BbTRAP2 is located in the apical end of the parasite and is a target for invasion inhibitory antibodies [18]. Like *B. bovis*, four *TRAP* genes also exist in *B. orientalis* and are considered as excellent vaccine candidates. BoTRAP1 has been identified which could be important for the interaction with ligands on the surface of the host cells and provide a theoretical basis for the discovery of novel *Babesia* vaccine candidate antigens [21].

Through long-term efforts, researchers in our laboratory have successfully constructed a cDNA library of *B. orientalis* and screened the *BoTRAP2* gene. The purpose of this study was to clone and express BoTRAP2 and to perform a bioinformatics analysis of this gene to elucidate its biological characteristics and potential molecular functions when invading the water buffalo, providing a candidate antigen for the further research of *Babesia*.

## Methods

### Experimental animals and strain

Three water buffalo were purchased from a *Babesia*-free area after confirming that they were free of *Babesia* by microscope examination and PCR. Two water buffalo were intrajugularly injected with the infected blood of *B. orientalis* (Wuhan strain) and bitten by infected *Rhipicephalus haemaphysaloides* after about two weeks of splenectomy. Blood samples were collected at a parasitaemia of ~3%.

### Genomic DNA and total RNA extraction

The leukocytes were removed before the DNA/RNA was extracted from the *B. orientalis*-infected erythrocytes in the blood. Genomic DNA was extracted from the *B. orientalis*-infected water buffalo blood using a TIANamp Genomic DNA Kit (Tiangen Biotech, Beijing, China) according to the manufacturer’s instructions and finally stored at −20 °C.

Total RNA was extracted from 400 μl of leukocyte-free *B. orientalis*-infected water buffalo blood by using a TRIzol^®^RNA(Invitrogen, CA, USA) following the manufacturer’s instructions. The RNA was converted into cDNA by reverse-transcribed PCR (RT-PCR) using a Fast Quant^®^ RT Kit (Tiangen Biotech), and then stored at −80 °C.

### Cloning and sequencing of BoTRAP2

The functional domain sequence of BoTRAP2 was amplified from both gDNA and cDNA with the specific primers (BoTRAP2-F/BoTRAP2-R) (Table 1). The thermal cycling parameters included the activation of Taq polymerase at 95 °C for 5 min, 35 cycles of (denaturation at 94 °C for 30 s, annealing at 62 °C for 30 s and extension at 72 °C for 45 s), and a final extension at 72 °C for 10 min.

**Table 1:** Primers used for the amplification of the partial BoTRAP2 genes

Primer	Primer sequence (5′–3′)	Restriction enzyme
BoTRAP2-F	ATAATGGACTTCGTGAGTTTGTTGAAG	
BoTRAP2-R	TACTGTTCGGACGATATTTATCGTATC	
BoTRAP2-1F	GAATTCGTGAGTTTGTTGAAGAACTGACTAAG	*Eco*RI
BoTRAP2-1R	CTCGAGAGGACCCAGAAGCTCTACCTTCAAC	*Xho*I

The truncated fragment of BoTRAP2 (BoTRAP2-1) contains vWFA and TSP domains. It was amplified from cDNA using the same PCR procedure as described above with specific primers (BoTRAP2-1-F/BoTRAP2-1-R) (Table 1).

The purity and length of the PCR products were checked on 0.8% agarose gel (Tsingke Biological Technology, Beijing, China), and then purified by an Easy Pure^®^ PCR Purification Kit (TransGen, Beijing, China). The fragments were ligated into a pEASY-Blunt vector (TaKaRa Biotechnology, Beijing, China) and then cloned into a pET-28a expression vector. All constructs were confirmed by DNA sequencing.

### Bioinformatics analysis

BoTRAP2-1 was analyzed by the conserved domain search service (CD Search) of NCBI (https://www.ncbi.nlm.nih.gov/Structure/cdd/wrpsb.cgi) for a better understanding of its vWFA and TSP1 domains. The putative TRAP nuclease sequences of *B. orientalis*, *B. bovis* (XP_001609812.1), *B. gibsoni* (BAI66058.1), *T. orientalis* (PVC51459.1), *P. vivax* (AAC97485.1), *N. caninum* (AAF01565.1) and *T. gondii* (XP_018637433.1) were compared by the MEGA7 software due to its advantage of multiple alignment [16, 19, 20, 22–24]. The diversity and evolutionary relationships of these sequences were explored by constructing a phylogenetic tree using the MEGA7 program [25]. The 3D-structure model of BoTRAP2-1 was subsequently predicted by SWISS-MODEL (https://swissmodel.expasy.org/interactive) according to the crystal structure of the proximal thread matrix protein 1 (PDB code: 4cn9.2.A) [26].

### Expression and purification of recombinant proteins

Sequences encoding the BoTRAP2 truncated fragment of *B. orientalis* were cloned and ligated into the expression vector pET-28a. The expression vectors were transformed separately into *E. coli* BL21 (DE3) strain, and the soluble proteins were purified using ProteinPure Ni-NTA Resin (TransGen Biotech) according to the manufacturer’s instructions after the induction of 1 mM IPTG (Biosharp, Anhui, China) overnight at 28 °C.

### Antibody generation

Anti-BoTRAP2-1 sera were raised in New Zealand white rabbits by five immunizations with a moderate amount of recombinant protein and Freund’s adjuvant (Sigma, Shanghai, China). The sera were collected when the serum titer reached an appropriate value. Total immunoglobulin Gs (IgGs) were purified from the collected sera by using a Protein A chromatography column (Beyotime Biotechnology, Shanghai, China) according to the manufacturer’s instructions and then stored at −20 °C.

### Preparation of *B. orientalis* lysates

The extraction of parasite protein was improved on the basis of a previous method [27]. Briefly, the erythrocytes were washed several times with phosphate-buffered saline (PBS), lysed with red blood cell lysate buffer (Tris/EDTA/NaCl), the precipitate collected by centrifugation at 13,000× rpm for 20 min, followed by several washes with PBS and storage at −20 °C. The extraction of water buffalo erythocyte membrane protein was performed in the same method. Finally, both of them were run on an SDS-PAGE gel and analyzed *via* western blot.

### Western blot analysis

In order to determine the immunogenicity of BoTRAP2, the protein of rBoTRAP2 was electrophoresed on 12% SDS-PAGE gels, then transferred into nitrocellulose membranes (Merck, NJ, USA) using the semi-dry blotting system and blocked with 5% (w/v) skimmed milk for at least 2 h or at 4 °C overnight. The nitrocellulose membranes were probed with both infected buffalo sera of *B. orientalis* and normal water buffalo sera. The secondary antibodies were BovIgG/HRP (1:2000) (Bioss, Beijing, China). After incubation for about 1 h, the bands were developed by the electro-chemi-luminescence (ECL) method.

Additionally, the native protein of BoTRAP2 was also confirmed by electrophoresing the products obtained from *B. orientalis* infected water buffalo erythrocytes and uninfected water buffalo erythrocytes on 12% SDS-PAGE gels and transferring them into nitrocellulose membranes following standard protocols. The primary antibody was rabbit polyclonal anti-rBoTRAP2 IgG and then probed with HRP labeled anti-rabbit IgG. After washing the membranes, the bands were developed by the ELC method.

### Indirect fluorescence assay

The air-dried thin smears of blood cells infected with *B. orientalis* at merozoite stages were fixed in cold 100% methanol (−20 °C) and permeabilized with 0.1% Triton X-100. Then, the blood smears were blocked with 5% FBS and incubated with a rabbit anti-BoTRAP2-1 specific IgG diluted 100 times with 1× PBS for 1 h at room temperature. The secondary antibody was goat anti-rabbit IgG (Alexa Fluor 488) diluted 1000 times with 1× PBS, followed by a parasite nucleus staining using hoechst stain for 1 h. Finally, 10 μl of anti-fluorescence quenching agent was used to prevent light quenching. The images were captured by a fluorescence microscope (Zeiss, Heidenheim, Germany).

## Results

### Molecular characterization and sequence analysis

**Fig. 1:**
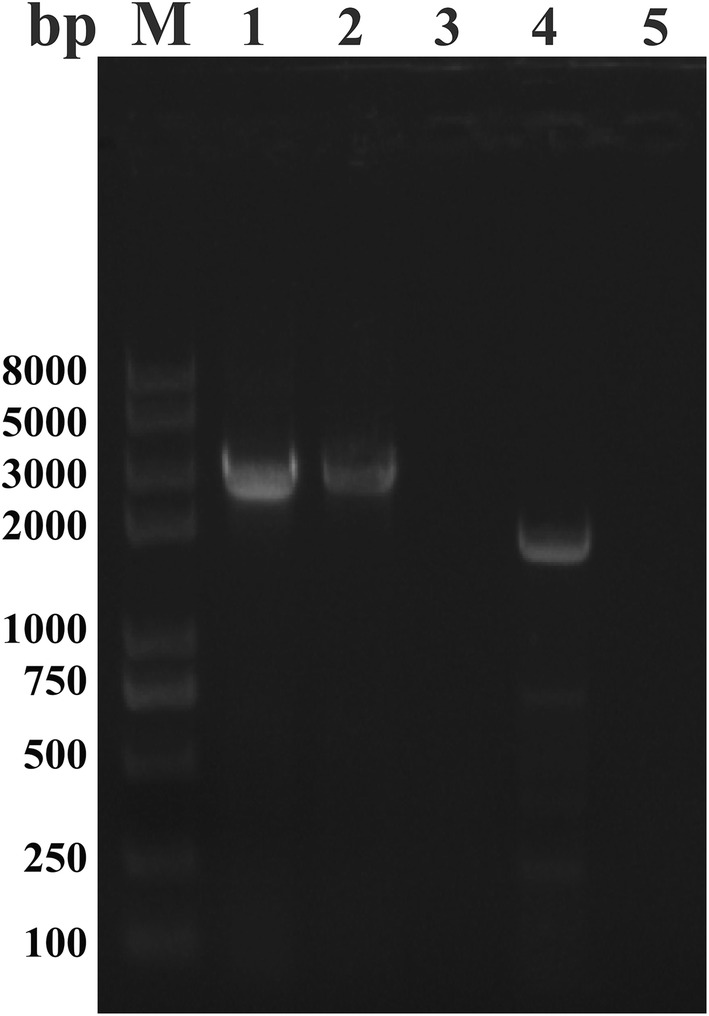
The PCR amplification of TRAP2 genes from gDNA and cDNA of *B. orientalis*. Lane M: marker; Lane 1: BoTRAP2 from gDNA; Lane 2: BoTRAP2 from cDNA; Lane 3: negative control; Lane 4: BoTRAP2-1 including two vWFA domains and one TSP domain; Lane 5: negative control

The functional domain of BoTRAP2 was amplified from both gDNA and cDNA by PCR using specific primers (Table 1). The sequence amplified from both gDNA and cDNA was 2817 bp without introns, encoding 938 amino acid residues (Fig. 1) with a predicted size 104 kDa. The BoTRAP2-1 amplified from cDNA was 2076 bp (Fig. 1) encoding 691 amino acid residues with a predicted size 77 kDa.

### Sequence analysis of BoTRAP2

**Fig. 2:**
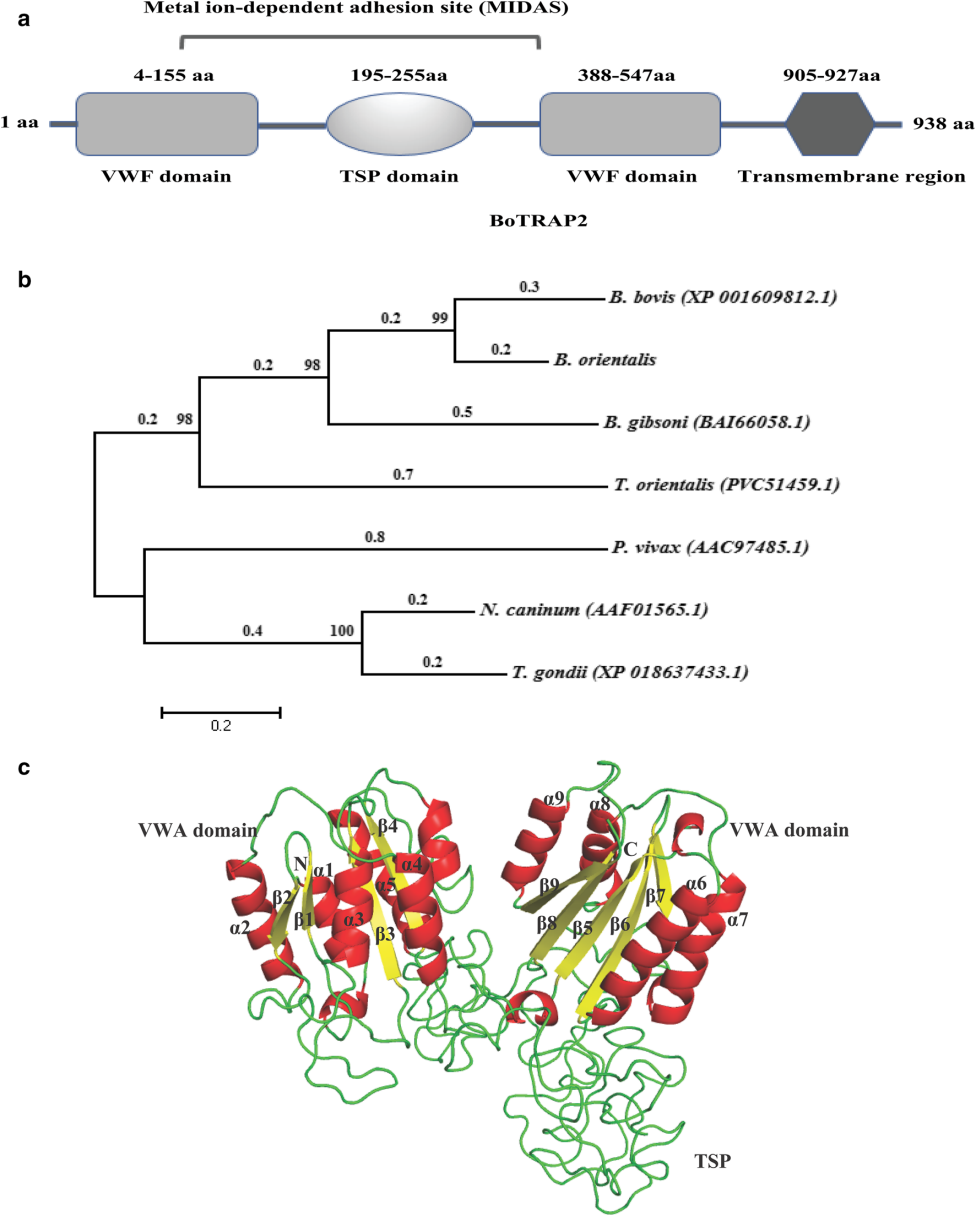
Sequence analysis of BoTRAP2. **a** Graphic depiction of the partial BoTRAP2 domains. The functional domains (vWFA and TSP) including MIDAS for interaction, followed by a 23-AA transmembrane region. **b** Neighbor-joining tree based on the BoTRAP2 sequence obtained in this study and the related apicomplexan parasite sequences. A bootstrap test of 1000 replicates was performed with the values given at the nodes. **c** The tertiary structure and channel of BoTRAP2 constructed by using the SWISS-MODEL. The 3D structure in identical orientations contains nine α-helices (α1 and α9) and nine β-strands (β1 to β9). The metal ion-dependent adhesion site (MIDAS) for binding protein ligands includes partial vWFA domain and TSP domain

The *BoTRAP2* gene has typical characteristics of the TRAP family, with a high similarity to the reported BbTRAP (XP_001609812.1) [20]. The BoTRAP2-1 with a vWFA domain, TSP-1 domain, and transmembrane domain was obtained by ligation into a cloning vector. The TRAP family proteins participate in important biological processes, such as vacuole membrane disruption and gamete egress from erythrocytes [28]. The amino acid sequence of BoTRAP2-1 was analyzed by using the BLAST tool on the NCBI website (https://www.ncbi.nlm.nih.gov). The results showed that the BoTRAP2-1 protein contains a typical TRAP-like family protein domain with a metal ion-dependent adhesion site (MIDAS), two vWFA domains and one TSP domain. Meanwhile, the vWFA domain and the TSP domain are arranged crosswise, as shown in Fig. 2a. The results demonstrate that the amplified BoTRAP2-1 is a member of the TRAP family.

The amino acid sequence of BoTRAP2-1 is 97 and 70% similar to that of *B. bovis* p18 (XP_001609812.1) and *B. gibsoni* TRAP (BAI66058.1), respectively. Phylogenetic trees based on the amplified BoTRAP2-1 and some other TRAP family protein sequences of piroplasms were constructed to provide a better understanding of the diversity of the sequences (Fig. 2b). The phylogenetic trees showed that *B. bovis* p18 was the closest clade to the BoTRAP2-1 sequence.

The tertiary structure of a protein refers to the arrangement of all atoms in the peptide chain in space. In our study, the tertiary structure of the BoTRAP2-1 protein was predicted by the Swiss Model software, indicating that the spatial simulation structure of the protein was composed of two chains consisting of 9 α-helices and 9 β-folds, with two vWFA domains, one TSP domain and MIDAS for interaction with ligands (Fig. 2c).

### Expression of the truncated BoTRAP2

**Fig. 3:**
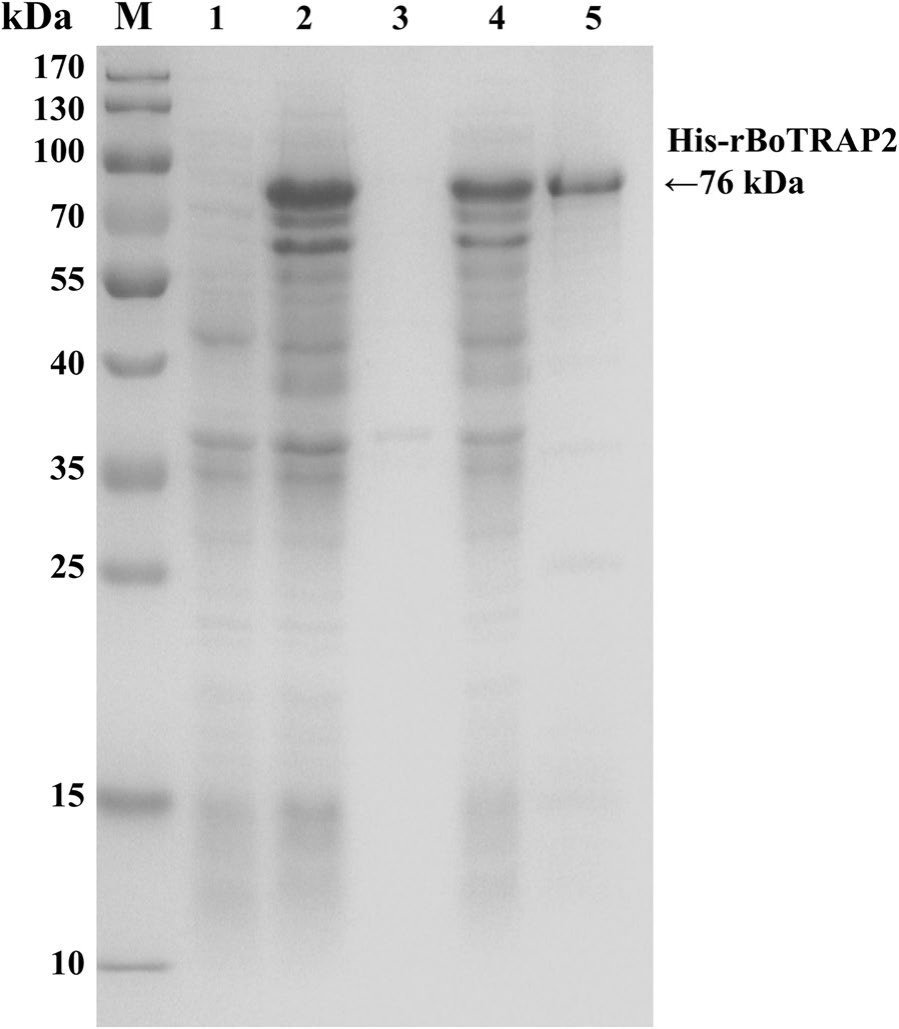
SDS-PAGE analysis of recombinant BoTRAP2. Lane 1: non-induced protein; Lane 2: induced control; Lane 3: precipitated his-BoTRAP2 in cell lysates; Lane 4: soluble his-BoTRAP2 in cell lysates; Lane 5: purified his-BoTRAP2

According to the results of sequence analysis, the correct plasmid was used to express the rBoTRAP2-1 protein containing a his-tag and purified by ProteinPure Ni-NTA Resin with a 77 kDa band (Fig. 3). Finally, the protein was collected and dialyzed to immune rabbits.

### Identification of the recombinant and native BoTRAP2 by Western blot

**Fig. 4:**
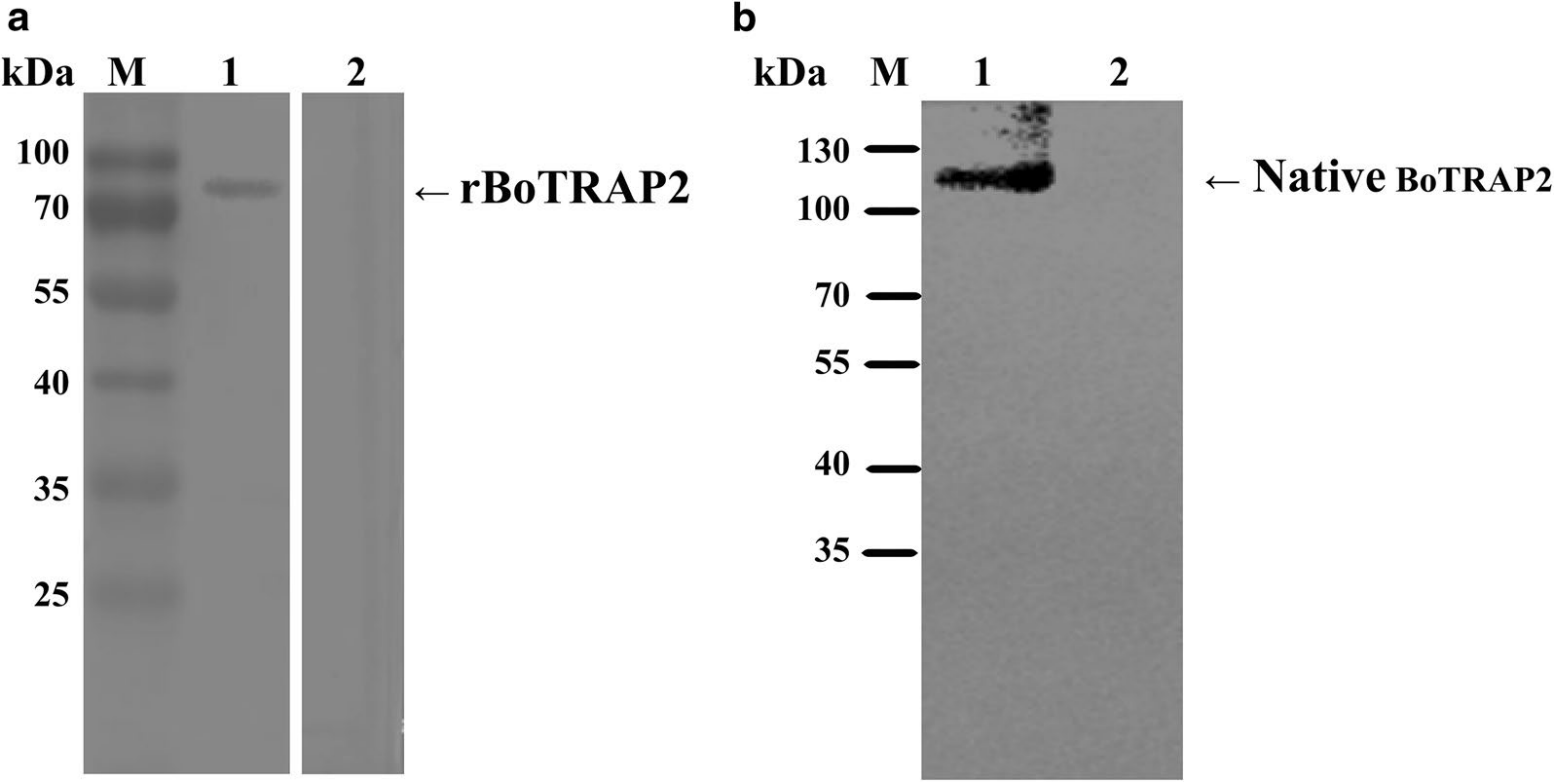
Western blot analysis of BoTRAP2. **a** Lane M: molecular weight marker; Lane 1: rBoTRAP2 reacted with *B. orientalis* positive serum; Lane 2: rBoTRAP2 probed with normal serum from water buffalo. **b** Identification of native BoTRAP2. Lane M: molecular weight marker; Lane 1: *B. orientalis* infected bovine RBCs lysates probed with rabbit anti-BoTRAP2 serum; Lane 2: non-infected bovine RBCs lysates probed with rabbit anti-BoTRAP2 serum

To identify the specific antigenicity of BoTRAP2-1, Western blot was performed through the reaction of rBoTRAP2-1 with the positive serum from the *B. orientalis*-infected buffalo, using the serum from the non-infected water buffalo as a control. The specific bands were detected from the *B. orientalis*-infected buffalo serum, but not from the control serum (Fig. 4a).

To identify the size of native BoTRAP2, the *B. orientalis* lysates were separately reacted with the purified anti-rBoTRAP2-1 IgG and the negative sera by using immunoblotting, while the lysate of non-infected RBCs was used as a control. Western blot results showed a band of ~110 kDa in the lysate of non-infected RBCs (Fig. 4b), but not in the lysate of normal RBCs. Nothing was detected on the immunoblotting membrane when using negative sera (data not shown).

### Localization of BoTRAP2

IFA was used to examine the subcellular localization of BoTRAP2 determined by rabbit anti-rBoTRAP2-1 polyclonal sera. The results showed that BoTRAP2 is localized on the apical end of the parasite as puncta protein specks (Additional file 1: Fig S1a, green) and the nuclear was stained by hoechst (Additional file 1: Fig S1a, blue), while no fluorescence was observed in the parasite with normal serum (Additional file 1: Fig S1b).

## Discussion

There are diverse mechanisms by which apicomplexan protozoa invade host cells, and the proteins involved in these processes include TRAPs [10]. All molecules that play important roles in these processes can be explored as potential candidates for drug development.

In this study, a gene encoding TRAP2 was cloned, purified and characterized in *B. orientalis*. Bioinformatics analysis showed that the BoTRAP2 sequence shared 97 and 70% similarity with that of the *TRAP2* gene of *B. bovis* and *B. gibsoni*, respectively, indicating that TRAP2 is a conserved protein in the *Babesia* genus (Fig. 1). Almost all TRAP family sequences contain one or more TSR domain, vWFA-A domain, TMD and CTD, except for the absence of the TSR domain from BbTRAP4 [7, 9, 18]. In immunoblotting analysis of the native BoTRAP2, a ~110 kDa band was revealed and the size corresponded to expected size. Compared with the size of BbTRAP2 (113 kDa), the size of native BoTRAP2 was most similar to those of BbTRAP2 [14, 16, 28]. The TRAP family is an important member of the apicomplexan membrane proteins. These proteins are rapidly transferred to the cell surface after parasite activation, possibly *via* Ca^2+^ signal transduction while they are stored in the micronemes [29]. This is consistent with the results of IFA.

The vWFA domain and TSR domain in an apicomplexan protozoan TRAP protein have been shown to be involved in the biological activities between cells and stroma, such as blood coagulation and innate immunity in parasites [30]. In *B. bovis*, the fusion-expressed BbTRAP2 protein could specifically recognize the positive serum collected from the *B. bovis*-infected cattle, and the thin smears of blood cells infected with *B. bovis* were probed with anti-BbTRAP2 serum for indirect fluorescence assay, which showed obvious fluorescence in the intracellular and extracellular parasites, respectively [18]. In apicomplexan protozoans, the TRAP proteins are involved in the invasion stage, and the invasion pattern in *B. bovis* has been elucidated [5]. During an asexual growth cycle of *Babesia* parasites in a natural host, the extracellular merozoites invade the host erythrocytes *via* various processes, such as attachment, penetration and internalization. Meanwhile, there are multiple adhesive interactions of several protozoan ligands in combination with the target receptors on the host cell surface. After internalization in the host erythrocytes, the parasites asexually propagate, then egress from the erythrocytes by rupturing the host cells, and invade a new RBC. In the initial attachment to the erythrocytes, various molecules of merozoites play essential roles in erythrocyte penetration or internalization, such as proteins secreted from apical organelles like micronemes. In *P. falciparum*, TRAP has been confirmed to form a tight complex with AMA1 and RON to adhere to the host erythrocytes [31].

In *Babesia gibsoni*, p18 was identified as a homologue of TRAP with a 70% similarity with BoTRAP2. Protein p18 consists of typical regions, including an SP, a vWFA domain, a TSP1 domain, a transmembrane region and a cytoplasmic C-terminus. As it is well known that TRAP protein has MIDAS sites in the vWFA domain, scholars considered that the protein on the erythrocyte membrane surface may bind this metal adhesion site. Subsequent studies on the adhesion of *B. gibsoni* p18 protein to erythrocyte membrane proteins denied this conjecture by adding different concentrations of Ca^2+^ [32]. This result suggested that the TRAP protein adheres to a certain protein on the host erythrocyte surface, rather than relying on polar charge attraction. In *Plasmodium* and *T. gondii*, the CTD region of the TRAP protein could form a tight junction with the host cell, which has also been identified to be a complex with actin under the action of aldolase. After successfully adhering to the molecules on the surface of the host cells, the parasites invade the host cells under the action of a dynamic system formed by aldolase and actin-myosin. The same phenomenon was confirmed in *B. gibsoni*, with the interaction between aldolase and BgTRAP observed by a pull-down assay [33].

## Conclusions

We obtained the open reading frame (ORF) of BoTRAP2 from both gDNA and cDNA with a length of 2817 bp without introns. The truncated fragment of BoTRAP2 was cloned, expressed with 77 kDa and confirmed to have great immunogenicity as a candidate antigen for the diagnosis of *B. orientalis*. The results showed that BoTRAP2 is localized on the apical end of the parasite. To our knowledge, this is the first report on the identification and characterization of BoTRAP2. Future studies should focus on the mechanisms of BoTRAP2 in the invasion into the host cells. Overall, BoTRAP2 could be a new molecular as a potential drug target for preventing and controlling *B. orientalis* in water buffalo.

## Additional file

**Additional file 1: Figure S1.** Localization of BoTRAP2 on *B. orientalis* by indirect immunofluorescence assay (IFA). **a** Polyclonal antibody and pre-immune serum. **b** Nuclei stained with Hoechst. Green indicates antibody reactivity and blue indicates parasite nuclei labeling. *Scale-bars*: 2 µm.

## Abbreviations

TRAP: thrombospondin-related anonymous protein
vWFA: von Willebrand factor A
TSR: thrombospondin type-I repeat
CTD: cytoplasmic tail domain
MIDAS: metal ion-dependent adhesion site
IPTG: isopropyl-β-d-thiogalactopyranoside
PBS: phosphate-buffered saline
RBC: red blood cell
