# Supplementary figures and images for: Identification of a novel thrombospondin-related anonymous protein (BoTRAP2) from Babesia orientalis
Source: Parasit Vectors. 2019 May 3;12:200. doi: 10.1186/s13071-019-3457-0 (PMC6500065; doi:10.1186/s13071-019-3457-0)

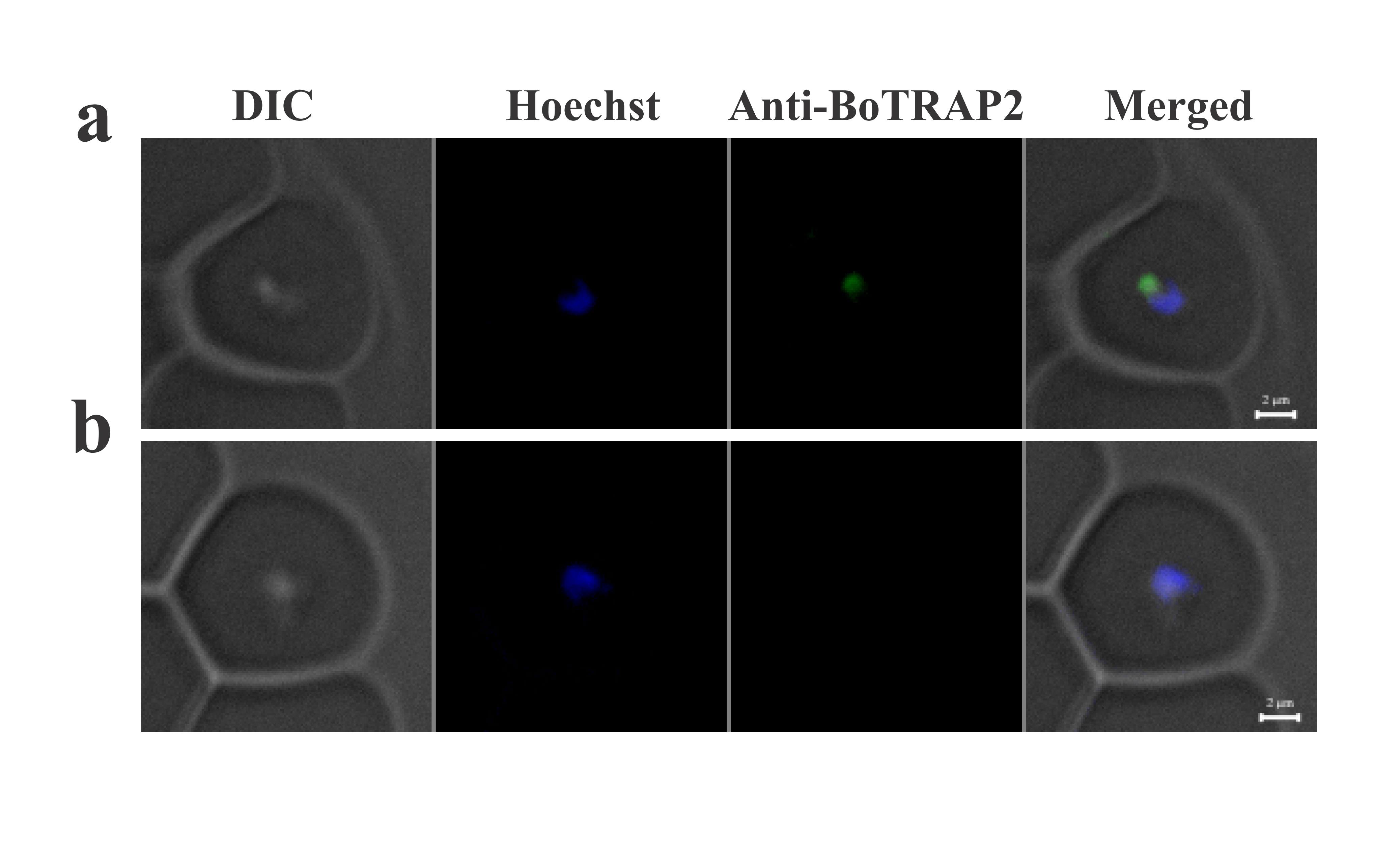

Supplement: Supplementary file 1 — Additional file 1: Figure S1. Localization of BoTRAP2 on B. orientalis by indirect immunofluorescence assay (IFA). a Polyclonal antibody and pre-immune serum. b Nuclei stained with Hoechst. Green indicates antibody reactivity and blue indicates parasite nuclei labeling. Scale-bars: 2 µm. [file 13071_2019_3457_MOESM1_ESM.tif]
